# Spermatic Vein Tumor Thrombus In Renal Cell Carcinoma

**DOI:** 10.1100/tsw.2004.67

**Published:** 2004-06-07

**Authors:** Nicola J. Mabjeesh, Yuval Bar-Yosef, Letizia Schreiber-Bramante, Issac Kaver, Haim Matzkin

**Affiliations:** Division of Urology, Tel Aviv Sourasky Medical Center, Tel Aviv, Israel

## Abstract

Renal cell carcinoma has the tendency to form venous thrombi. This may involve the renal veins or the inferior vena cava and may extend cephalad/antegrade into the right atrium. We report a patient with renal cell carcinoma who had an intracaval tumor thrombus that had extended into the right spermatic vein. We believe this to be the first description in English literature of a histologically proven renal cell carcinoma thrombus in the spermatic vein.

## CASE REPORT

A 64-year-old man was referred to our department due to a large right renal tumor. During the physical examination, a large solid mass was palpated on the right abdomen. Computerized tomography showed a tumor in the right kidney that extended into the inferior vena cava. There was no evidence of distant metastases; all laboratory values were within normal limits except for hemoglobin, which was 9.5 gr./dL (normal range: 11.7-17 gr./dL). Doppler ultrasonography revealed a tumor thrombus within the inferior vena cava that extended upward to the level of the hepatic veins and reached 4 cm below the level of the diaphragm.

A preoperative arteriographic embolization of the right renal arteries was completed. The operation was performed through a right thoracoabdominal approach. The renal tumor had invaded the third and fourth part of the duodenum, but no lymph node enlargement was noted. Radical nephrectomy was then performed, including extraction of the tumor thrombus from the inferior vena cava. A dilated right spermatic vein was detected during the dissection of the inferomedial part of Gerota¹s fascia. It was palpated and found to contain a tumor thrombus (fig. 1). The vein was ligated and disconnected at its insertion into the inferior vena cava. After confirmation by means of a Fogarty catheter that no thrombus was left distally, the vein was resected at the level of the inguinal canal. The postoperative course was unremarkable.

Microscopic examination showed a large renal cell carcinoma that was of clear cell type, extensively necrotic and infiltrating into the entire kidney and the perinephric fat, including the renal vein, inferior vena cava and spermatic vein (fig. 2). A part of the duodenal wall was also involved with tumor.

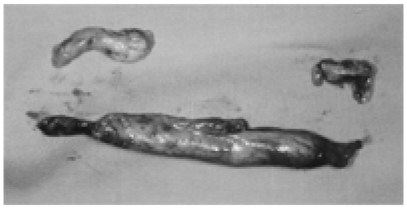

**FIGURE 1**. The right spermatic vein with the tumor thrombus. A part of the tumor thrombus that was extracted from the distal spermatic vein can also be seen.

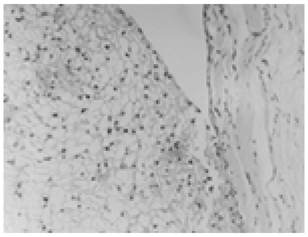

**FIGURE 2.** Light microscopy showing intraluminal renal cell carcinoma of the spermatic vein. H & E, magnification x20.

## DISCUSSION

The phenomenon of renal cell carcinoma invading and extending into the renal veins as a tumor thrombus is well recognized. The thrombus may involve the intra-renal veins, the main renal veins, the inferior vena cava and extend into the right atrium. Continued growth of the thrombus into the vena cava occurs in a small number of cases, and usually without direct invasion of the vessel. Since the recognition of the importance of caval extension and aggressive surgical treatment, however, it is understood that most patients can still be cured surgically. Metastatic tumor to the spermatic vein from renal cell carcinoma is unusual, with only five cases having been described in Japanese literature1 and one in French.2 The pathophysiological mechanism for this rare retrograde neoplastic extension to the spermatic vein is not yet defined. It is believed that tumor thrombus generally extends in accordance with the venous blood flow (from the intra-renal veins upto the right atrium). In our case of spermatic vein involvement, however, the thrombus would have had to spread in a retrograde fashion and grow against the normal venous blood flow. Our patient did not have any preoperative clinical or radiographic signs of spermatic vein involvement. The diagnosis was done intraoperatively. It is not clear what the consequences of such tumor extension are in terms of prognosis, or whether the tumor¹s resection would be of prognostic benefit. Given the advanced stage (T4) of disease of our patient, it would be especially difficult to determine the prognostic impact of spermatic vein involvement with much accuracy. In our present case, the spermatic vein and its content were intuitively resected following the aggressive surgical principles of achieving a cure. We also believe that in future cases of large renal tumors with extension to vena cava, it might be worthy to further evaluate the spermatic veins for tumor thrombi, or even perform ultrasonography of the ipsilateral testis when a thrombus is found within the spermatic vein.
